# Mental health support for and telehealth use by Australians living with borderline personality disorder during the onset of the COVID-19 pandemic: A national study

**DOI:** 10.1177/20552076231169824

**Published:** 2023-05-04

**Authors:** Parvaneh Heidari, Jillian H Broadbear, Rita Brown, Nitin P Dharwadkar, Sathya Rao

**Affiliations:** 1 Spectrum Personality Disorder and Complex Trauma Service, 1890 Eastern Health, Richmond, Victoria, Australia; 2 Eastern Health Clinical School, Monash University, Clayton, Victoria, Australia; 3 Personality Disorder & Complex Trauma Research and Innovation Centre, Richmond, Victoria, Australia; 4 Australian BPD Foundation Ltd, Bayswater, Victoria, Australia; 5 Department of Psychiatry, Central Clinical School, Monash University, Melbourne, Victoria, Australia; 6 School of Clinical Sciences, Monash University, Clayton, Victoria, Australia

**Keywords:** Australia, borderline personality disorder, COVID-19 pandemic, personality disorder, telehealth, telemedicine

## Abstract

**Objective:**

To investigate mental health service use and telehealth experience of people living with BPD in Australia during the first year of the COVID-19 pandemic.

**Methods:**

An online survey was used to collect data from people who self-identified with a diagnosis of BPD.

**Results:**

One hundred and sixty-nine survey responses were included in the analysis. More than half of participants acknowledged receiving information from their health service about resources that they could use if they become distressed. More than 70% of participants used telehealth for receiving mental health services; the majority used telehealth to consult a psychologist or to obtain prescriptions. Telehealth sessions were conducted over the phone, via videoconferencing, or using a mix of the two. While using telehealth, some participants found it more difficult to control their impulses to self-harm, to express thoughts about self-harm and suicide, to control feelings of anger, and to establish and maintain agreed treatment boundaries. Thematic analysis of participants’ experiences of telehealth identified five main themes: Communication challenges, Technology challenges, Privacy concerns, Benefits of telehealth, and Personal preferences.

**Conclusion:**

The study findings revealed a variety of positive and negative consumer experiences. While the majority of participants found telehealth somewhat benefitted their mental health, challenges were also reported which raise concerns about the broader utility and effectiveness of telehealth.

## Introduction

Evidence-based psychotherapy treatments are proven to help many people living with borderline personality disorder (BPD) to achieve sustained symptomatic remission.^1,2^ The most common mode for delivering these psychotherapeutic treatments is in-person (through outpatient or residential programmes). For the treatment of mental health difficulties more broadly, some evidence suggests that the use of telehealth is generally acceptable and comparable to in-person treatment.^3^ A pre-pandemic Australian review that investigated clients’ preferences found a preference for in-person treatments over telehealth.^4^ A during-pandemic study based in the United Kingdom found that mental health clients’ use of telehealth was shaped by their reason for contacting services, their relationship with care providers, access to technology, and their individual preferences. Although in-person was their preferred mode of care, clients acknowledged benefits associated with telehealth including improved access and efficiency for some appointments, such as prescription reviews. Clients highlighted challenges related to maintaining privacy and safety in telehealth, and provided examples of good telehealth strategies they had experienced, including the scheduling of regular phone calls and providing guidance for accessing telehealth tools.^5^

BPD may be experienced as intense emotional reactivity, troubled interpersonal relationships, and an unstable sense of self-concept.^6,7^ BPD is also associated with frequent episodes of non-suicidal self-harm, suicidality, emptiness, anger, impulsive behaviour, and dissociative symptoms.^7^ BPD is a complex and serious mental disorder which has the capacity to dramatically undermine a person's well-being and life expectancy.^8^ A survey conducted among clients of a specialised clinic for personality disorder reported that many experienced significant challenges to their overall well-being during COVID-related lockdowns.^9^ The high levels of distress, day-to-day challenges, and social isolation experienced by people living with BPD highlights the importance of understanding the experience of telehealth in this vulnerable population.

Some recent studies have investigated the utility of telehealth in people with BPD during the pandemic from a clinician perspective. A US study was in a partial hospitalisation setting, which provides a transition from an inpatient admission to facilitate stabilisation and skill acquisition in preparation for discharge into the community). During the pandemic, when these in-person transition services moved to telehealth, the study reported that telehealth treatment was as effective as in-person treatment in terms of symptom reduction, patient satisfaction, and improved functioning and well-being.^10^ An Australian study, conducted during the first year of social restrictions, described the experiences of mental health clinicians who used telehealth platforms to deliver individual and group psychotherapy to clients diagnosed with BPD.^11^ They reported benefits of increased flexibility when scheduling appointments, finding that clients attended more consistently than when they were previously offered in-person treatment. However, the clinicians raised concerns about privacy, confidentiality, unmanaged clinical risk, reduced quality of interaction, and difficulty maintaining treatment boundaries. Despite these concerns, none of the clinicians reported any suicide-related incidents during the time that they were exclusively using telehealth.^11^ In another Australian study, 18 out of 28 (64%) clinicians stated they had low or no confidence when delivering psychotherapy via telehealth.^12^ Although almost half (45%) reported that the quality of their therapeutic relationship with their clients was about the same as before, 54% reported that their relationship was worse or a lot worse when using telehealth than it had been during in-person interactions.^12^

When considering the perspectives of consumers with BPD, an Australian survey of 37 clients engaged in treatment at a specialist personality disorder service found that only two (5.4%) discontinued treatment following the transition to telehealth during the first wave of COVID-19 social restrictions. Clients reported positive and negative experiences of telehealth, with about 50% acknowledging the importance of telehealth and keen to retain the option of using telehealth post-pandemic.^13^ A qualitative study in specialist mental health services located in Norway reported that participants with personality disorder had diverse feelings and experiences with using telehealth. Two meta-themes were reported. One comprised themes that reflected positive aspects of telehealth: participants felt relieved that using telehealth protected them from the virus, provided a platform where they could be sincere and genuine, and provided an emergency solution. They also acknowledged that frequent and predictable therapist contact reduced insecurity and loneliness. However the other meta-theme included negative experiences, including the sense that telehealth brought an emotional distance to therapy due to technical challenges associated with the platform, less profound conversation in therapy sessions, and concerns around privacy.^14^ To our knowledge, no community-wide study has investigated the mental health service use and telehealth experience of people with a diagnosis of BPD. Given the high likelihood that telehealth-based treatment and services will continue to be available post-pandemic, learning from the experiences of this vulnerable population will be beneficial for planning future interventions. Therefore the aim of this study is to investigate mental health service use and telehealth experiences of community-dwelling Australians living with BPD during the first year of the COVID-19 pandemic.

## Methods

This study collected quantitative (multiple-choice questions) and qualitative data (open-ended questions) using a convergent parallel mixed-method design. The survey was live during July and August of 2020 to capture experiences following the first national lockdown due to the global COVID-19 pandemic. People residing in Australia who self-identified with a diagnosis of BPD were invited to participate. The study received approval from the institutional human research ethics committee (LR20/037).

### Survey

The mixed-methods survey (closed- and open-ended questions) was developed for this study by the study authors and revised following consultation with mental health clinicians and a lived experience carer consultant. The first section of the survey included screening questions about age, geographic location, and the person's identification with having a diagnosis of BPD. The study exclusion criteria were: being under 18 years of age, residing outside Australia, or not having received or identifying with a diagnosis of BPD. If any of the exclusion criteria were endorsed, respondents were directed to the end of the survey. Subsequent survey questions enquired about:

- Demographic characteristics such as gender, the state or territory where participants resided, who they resided with, and their employment status,
- Telehealth use and experience, and
- Participants’ personal well-being during the social isolation restrictions

The survey comprised 60 questions; the average time spent completing the survey was 16 minutes. This article reports the findings in relation to mental health service use and telehealth experiences. The survey findings regarding participant well-being are reported separately.

### Recruitment

The study survey enabled participants to anonymously enter information about themselves on a secure online platform (Survey Monkey^TM^). Completion of the survey was voluntary and consent was implied if a participant engaged with the survey questions. The study invitation and survey link were promoted via social media platforms including a BPD specialist clinic website, LinkedIn, Twitter, and the Australian BPD Foundation website and monthly newsletter.

### Analysis

Quantitative data were analysed using descriptive frequencies and percentages. Statistical analyses were carried out using Statistical Package for the Social Sciences (SPSS; version 25). Chi-square tests for independence were conducted to examine whether differences in the demographic characteristics of participants (age, location, and gender) influenced the (i) preference for telehealth in comparison with in-person services and (ii) perceived accessibility of mental health services during the social restrictions. The threshold for statistical significance was set at 5%. Inductive thematic analysis described by Braun and Clarke (2006)^15^ was used to identify themes related to telehealth experience from the qualitative data arising from the open-ended questions. Qualitative data were imported into NVivo (QSR International). Two researchers (PH and JB) independently read and re-read the text and coded the responses of six randomly selected participants. Discrepancies in coding were discussed and a coding framework was developed. New codes were added when novel themes were identified when coding the remaining participant responses. PH and JB reached consensus decisions after all data were coded.

## Results

In total 226 surveys were submitted; 169 surveys were included in the study analysis. Survey responses were excluded if more than half of the questions had not been answered. Demographic characteristics of participants are presented in the supplementary material. The majority of participants were female (89.9%), resided in the most populous states of Victoria or New South Wales (63.9%) and were aged between 28 and 47 years (58.6%). Thirty-nine participants (23.1%) lived alone. Depression and anxiety were the most common comorbid mental health disorders. In terms of employment, 28.4% of participants reported being unemployed prior to the pandemic.

### Service use and information needs

The most common mental health service that participants were accessing prior to the pandemic was in-person treatment from a private psychologist (Supplementary material and Table 1). Overall, mental health service use was low among study participants, particularly their use of public mental health services (Table 1). Participants who were accessing mental health services prior to the pandemic reported that the majority of these mental health services continued to be provided in-person or via telehealth during lockdown, with only a few ceasing altogether after restrictions began (Table 1). More than half the participants stated that they did not receive information about accessing (alternative) supports and services during the restrictions. They noted that receiving information about helpful resources to access if they become distressed would have been very useful (Table 2).

**Table 1.** Availability of mental health services for Australians living with BPD during the COVID-19 pandemic restrictions.

Service type (number of responses)	*n* (%)			
	Yes, they offered in-person service	Yes, they offered telehealth service	No, they stopped offering this service	I was not using this service before the start of restrictions
Prevention and Recovery Care (PARC) (*n* = 132)	7 (5.3)	5 (3.7)	5 (3.7)	115 (87.1)
Crisis Assessment and Treatment Team (CATT) (*n* = 134)	10 (7.4)	12 (8.9)	8 (5.9)	104 (77.6)
Drug and Alcohol Services (*n* = 129)	4 (3.1)	5 (3.8)	1 (0.7)	119 (92.2)
Private psychologist (*n* = 150)	24 (16.0)	58 (38.6)	4 (2.6)	64 (42.6)
Private psychiatrist (*n* = 142)	16 (11.2)	37 (26.0)	3 (2.1)	86 (60.5)
Case management (*n* = 135)	10 (7.4)	16 (11.8)	7 (5.1)	102 (75.5)
Public psychologist (*n* = 135)	11 (8.1)	18 (13.3)	6 (4.4)	100 (74.0)
Public psychiatrist (*n* = 128)	12 (9.3)	7 (5.4)	4 (3.1)	105 (82.0)
Mental health nurse via GP Clinic (*n* = 128)	8 (6.2)	9 (7.0)	1 (0.7)	110 (85.9)

BPD: borderline personality disorder.

**Table 2.** Information and support provided to Australians living with BPD during the COVID-19 pandemic restrictions.

	*N*	%
**Were you provided with information and/or access to (alternative) supports and services during the restrictions? (*n* = 168)**		
Yes	45	26.8
No	92	54.8
Sometimes	31	18.5
**Do you feel that any information/support you were provided was appropriate for meeting your needs? (*n* = 167)**		
Yes	34	20.4
Partially	62	37.1
No	46	27.5
Not applicable	25	15.0
**What additional telehealth supports would you have found helpful when in-person services were no longer available? (*n* = 78)**		
Initial training from my service provider in the use of telehealth	12	15.3
Support with the purchase and setting up of equipment to use for accessing telehealth services	15	19.2
Support with improving my internet access	15	19.2
Information about resources that I could use if I became distressed when accessing services via telehealth	52	66.6
Clarity regarding privacy and confidentiality of telehealth consultations	36	46.1

BPD: borderline personality disorder.

### Telehealth

**Figure 1.**
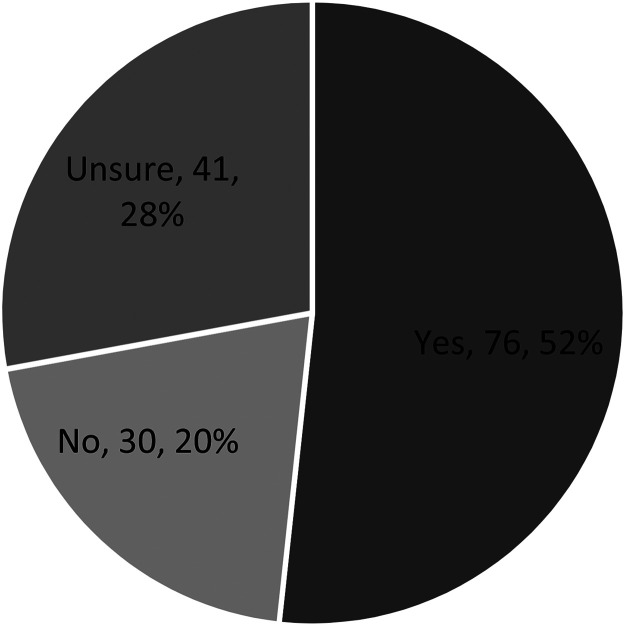
Responses to participants being asked whether they would like to have the option of receiving mental health services via telehealth in the future (*n* = 147).

The majority of participants (70.4%) accessed telehealth services to obtain support for some aspect(s) of their mental health during the restrictions. The majority of these participants used telehealth to consult a psychologist or obtain prescriptions (Table 3). When asked about the process of transitioning from in-person services to telehealth, most reported not experiencing any major problems (66.6%). Table 3 summarises participants’ experiences using telehealth during the pandemic, including problems accessing the internet, concerns regarding privacy, and the helpfulness and accessibility of services. Most participants used a mix of phone and videoconferencing, with 31% using only phone and 17% using only videoconferencing. There were no statistically significant differences between the demographic characteristics of participants (age, location, and gender) and (i) preference for telehealth compared with in-person services (*p *> 0.05), and (ii) perceived accessibility of mental health services during the social restrictions (*p *> 0.05) (Table 3). Among participants who only used phone, the most commonly reported reason was that their service provider did not offer a videoconference option (Table 4). In terms of the consumer experience among participants who used only videoconferencing, the majority reported that they sometimes or always experienced social pressure, distraction, self-consciousness, anxiety and insecurity (Table 5). More than half of all participants expressed the desire to retain the option of telehealth services for future mental health needs (Figure 1).

**Table 3.** Experience of using telehealth to access mental health services during the pandemic by Australians living with BPD.

	*n*	%
**Did you use telehealth for any mental health service during the restrictions? (*n* = 169) **	119	70.4
**What telehe** ****alth se**rvices did you use? (participants responding 'yes' to previous question, *n* = 113)**		
Obtaining prescriptions	58	51.3
Sessions with a psychiatrist	40	35.4
Sessions with a psychologist	76	67.3
Sessions with a mental health nurse, support worker or similar	26	23.0
Crisis support (e.g., Crisis Assessment and Treatment Team; CATT)	13	11.5
Case Management	19	16.8
Psychotherapy (individual and/or group)	18	15.9
**From a technical point of view (e.g. access to equipment, learning how to connect, stability of connection), how did you experience the transition from in-person service to telehealth? (*n* = 114)**		
I experienced few or no technical issues with the shift to telehealth	51	44.7
It was a bit chaotic at first getting the technology working but I succeeded after a few tries	25	21.9
It was a bumpy transition with ongoing technical problems	13	11.4
It was so difficult to get the sound and video working that I just used the telephone	10	8.8
I decided that I could not communicate via telehealth, so my mental health support went on hold during the restrictions	9	7.9
Has limited internet access (perhaps due to cost, lack of suitable devices, or internet connection/plan) influenced your decision about whether and how to use telehealth? (*n* = 114)		
Yes	25	21.9
Sometimes	25	21.9
No	64	56.1
**Do you have any concerns regarding privacy when using telehealth? (*n* = 114)**		
Yes	25	21.9
Sometimes	23	20.2
No	66	57.9
**How helpful has accessing services via telehealth been for you? (*n* = 114)**		
Extremely helpful	18	15.8
Very helpful	22	19.3
Moderately helpful	37	32.5
Sometimes helpful	27	23.7
Not at all helpful	10	8.8
Telehealth support that I received during the restrictions was conducted: (*n* = 111)		
Over the phone only	35	31.5
Using videoconferencing only	19	17.1
Using a mix of phone and videoconferencing depending on what service was being provided	45	40.5
The mode of telehealth delivery varied depending on the clinician/provider	12	10.8
**Are there aspects of telehealth that worked better for you compared with when you receive in-person psychological treatment? (*n* = 99)**		
Yes	36	36.3
No	46	46.4
Unsure	8	8.0
I'm not currently receiving psychological treatment	9	9.0
**How accessible do you feel that your mental health service provider(s) was/were during the restrictions compared to when you were meeting in-person? (*n* = 99)**		
More accessible	11	11.1
About the same	57	57.5
Less accessible	31	31.3

BPD: borderline personality disorder.

**Table 4.** Reasons for Australians living with BPD using telephone to receive mental health services during the pandemic (*n* = 43).

Experiences of obtaining telehealth services via phone	*N*	%
**If you used phone only, what led you to choose that telehealth format?**		
Technical problems getting audio and video both working meant that phone consultations were the best option	7	16.3
Privacy concerns (e.g., phone conversation is more private than using a device with a screen that others can see)	4	9.3
I found that video telehealth created more social pressure and anxiety	13	30.2
I found that video telehealth intruded into my personal space/life	8	18.6
I found it distracting being able to see a moving image of myself on the screen during a session	7	16.3
I felt self-conscious or anxious about my appearance during video telehealth consultation	14	32.6
I felt more insecure having to see my own face and facial expressions over the course of the session	13	30.2
The video option was not offered by the service provider	17	39.5
No particular reason. It is just easier to have a phone call.	8	18.6
Other	8	18.6

BPD: borderline personality disorder.

**Table 5.** Experience of Australians living with BPD using videoconferencing to receive mental health service during the pandemic (*n* = 71).

Choose which of the following options apply to your experience of using videoconferencing	Yes		Sometimes		No	
	*n*	%	*n*	%	*n*	%
I found that video telehealth created more social pressure and anxiety	21	29.6	24	33.8	26	36.6
I found that video telehealth intruded into my personal space/life	17	24.3	19	27.1	34	48.6
I found it distracting being able to see a moving image of myself on the screen during a session	28	40.0	18	25.7	24	34.3
I found it more relaxing being in a familiar home environment	18	25.7	26	37.1	26	37.1
I felt self-conscious or anxious about my appearance during the video telehealth consultation	29	40.8	25	35.2	17	23.9
I felt more insecure having to see my own face and facial expressions over the course of the session	29	41.4	26	37.1	15	21.4
I liked being able to see my clinician in a non-clinical setting (if they were using telehealth from home)	25	36.2	20	28.9	24	34.7

BPD: borderline personality disorder.

### Therapeutic aspects of using telehealth

**Figure 2.**
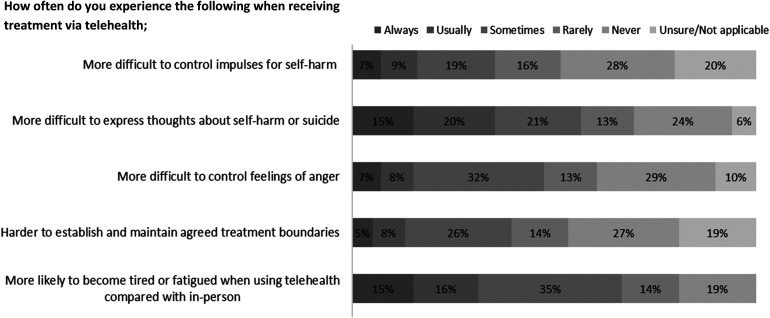
Ability to manage difficult thoughts and feelings when receiving treatment via telehealth in comparison with in-person treatment (*n* = 99).

When asked whether using telehealth led to any changes in the way participants practice previously learned coping skills and strategies, the majority (*n* = 99) answered ‘no’ (*n* = 45, 45.4%). The rest were 'unsure' (n=27, 27.2%) or answered 'yes' (n=27, 27.2%). Within the context of treatment sessions accessed via telehealth, Figure 2 illustrates how participants felt that telehealth affected their ability to communicate with their clinician. Table 6 reports whether participants living with BPD were able to manage difficult conversations when communicating with their clinician via telehealth.

**Table 6.** Experiences with regard to managing difficult conversations when communicating with aclinician via telehealth (*n* = 99).

	*n*	%
It was often easier to have difficult conversations when we were not meeting in-person	17	17.1
I found that telehealth is no different from in-person for managing difficult conversations	17	17.1
Difficult conversations were more likely as it was easier to misunderstand each other when communicating via telehealth	15	15.1
Therapy often involves difficult conversations however these were harder to resolve over telehealth	33	33.3
If conversations get difficult it is easier for me to choose not to engage when we are communicating via telehealth	34	34.3
If conversations get difficult I can leave the conversation more easily when using telehealth	22	22.2
I avoided difficult topics during telehealth sessions because of privacy concerns (i.e. I may be overheard; lack of security of the telehealth connection)	24	24.2

BPD: borderline personality disorder.

### Qualitative results

The thematic analysis of participants’ experiences of telehealth identified five main themes: Communication challenges, Technology challenges, Privacy concerns, Benefits of telehealth, and Personal preferences (Table 7).

**Table 7.** Qualitative results; experience of Australians living with BPD when using telehealth for mental health services.

Theme	Categories	Representative quotes
**Communication challenges**	Staying focused	‘It takes a lot more concentration to stay focused and pay attention to what is being discussed with telehealth’
	Missing the in-person human communication and feeling isolated	‘Went from in-person therapy to online and I missed the human contact and having a reason to get out of the house’
	Body language is missed	‘People can't see your state/body language‘
**Technology challenges**	Poor quality of internet connection: participants stated they either had cheaper internet packages that had lower connection quality, internet was disconnected in their area for several days, or poor quality of internet generally.	‘The WiFi network struggled with the increased demand and dropped out repeatedly.’
	Poor access to/quality of equipment	‘Not having access to a desktop made telehealth less appealing because I had to use phone’
	Health provider was not flexible with the videoconferencing platform	‘I could not access the video app that the GP was allowed to use so we had to do phone which is not great’
	Health provider only offered phone calls	‘I didn’t get a choice, my GP would only do phone. I can’t speak on the phone due to anxiety and past trauma so it made my experience worse and cut me off of health and support.’
	Not knowing or not interested in learning the skills relating to telehealth use	‘Drop out because I didn't know how to make it work.’
**Privacy concerns**	Other people in the clinician's home can hear	‘Sometimes I wonder who else is in their room’
	Private space for clinician	‘I was on a telehealth consult with an OT when the computer was accidently moved and I saw there was someone sitting just out of view from the screen that I didn’t know about.’
	Private space for the participant	‘Very hard to undertake telehealth services with family in home too’
	Session is being recorded	‘May be audio/screen recorders active, easy to hack, easier to hack professional files.’
	No privacy policy was provided	‘The privacy policy is not easily available for me to read’
**Benefits of telehealth**	Worked well with telehealth	‘I am able to express myself better over the phone than face to face so it's a positive for me. As long as I am aware of when the practitioner will go so I can prepare.’
	Time flexibility	‘Without Telehealth, I would not be able to schedule my appointments around work needs.’
	No travel costs	‘I didn't have to worry about money for travel. That was a plus. But like I said, I prefer to see a therapist in person.’
	Less stress to leave home	‘Less stress and anxiety to leave home to attend the appointment or group DBT’
	Feel more independent and empowered	‘I did a lot of the work myself rather than relying on a therapist. This was great.’
	Comfortable at homes	‘Location/travel was more preferable as I was home. Being with my animals when in session helped me cope with difficult topics.’
**Personal preferences**	Telehealth is fine but in-person is better	‘I'm not sure … I feel like in-person care helps me more. Over the phone has been a help during the isolation though.’
	Prefer in-person service	‘I only used phone consultation to request referrals or prescriptions. I wouldn’t be comfortable using it for anything more serious or via video’

BPD: borderline personality disorder.

## Discussion

This study is the first nationally focussed community study to investigate the telehealth experience of people living with BPD during the COVID-19 pandemic. Most (91%) participants reported that telehealth benefitted their mental health (35% found it very or extremely helpful); only 8.8% stated that it was not at all helpful. More than half wanted to retain the option of using telehealth for future mental health services. Despite this mostly positive appraisal, participants consistently affirmed that in-person treatment was more beneficial for helping them with their mental health difficulties. This is consistent with the findings of previous studies in mental health^5^ and personality disorder^14^ clients, who acknowledged telehealth benefits during the pandemic but maintained their preference for in-person mental health treatment. A pre-pandemic study also reported the same preference,^4^ One difference that emerged in studies conducted during the pandemic was the high endorsement of telehealth services: mental health clients in general acknowledged the improved access and efficiency for appointments such as prescription reviews;^5^ personality disorder clients in particular acknowledged that telehealth reduced insecurity and loneliness during the restrictions, in addition to other aforementioned benefits.^14^ Previous studies investigating the clinical effectiveness of receiving BPD treatment via email^16^ or using virtual formats^17^ reported that this was as effective as in-person treatment. For example, in their non-randomised clinical trial, Alavi and colleagues^16^ found no significant differences in treatment efficacy between an email-based format and an in-person group format for delivery of a dialectical behavioural therapy skill-building programme.^16^ This is somewhat unexpected given the findings reported in the present study. It is possible that the non-randomised allocation of delivery format by Alavi and colleagues’^16^participant personal preference, highlighting the importance of choice in the effectiveness of service delivery. In the present cross-sectional study, participants were at different stages of their recovery journey when in-person services switched to telehealth. These differences were apparent in some of the open-text responses, where a subset of participants who may have been further along in treatment reported that using telehealth provided an opportunity for them to practice learned skills and did not affect the quality of interaction with their therapist.

This study highlights some of the benefits and challenges associated with the use of telehealth to deliver mental health services for people living with BPD. An earlier study investigating the telehealth experiences of clients of a specialist clinic for personality disorders also endorsed positive and negative experiences.^13^ The experiences ofclients engaged with a specialist service appear to generalise to the community sample reported in the present study. This was evident in the expressed feelings of insecurity, self-consciousness, anxiety, or distraction while using videoconferencing platforms. Both studies also agreed with respect to challenges of staying engaged and focused during telehealth sessions. Issues related to receiving treatment via telehealth are not restricted to people diagnosed with BPD, and appear to generalise to other mental health disorders.^18^ A commonly reported challenge is having unequal access to telehealth technology. Concerns around confidentiality, privacy, and developing a therapeutic relationship were also reported.^18^ While there are considerable advantages to telehealth such as accessibility, not having to travel (time and costs), and feeling comfortable in a familiar setting, these must be balanced with prominent challenges in relation to access to technology, privacy, and quality of communication. The very rapid and mostly unplanned transition from in-person services to telehealth service delivery likely contributed to participant perceptions of the inadequate provision of resourcing and information to support their mental health needs. The lack of consistent guidelines and privacy policies, and unavailability of technical support for mental health consumers at this time, was also notable.

The findings of this study are likely to assist mental health clinicians, healthcare managers, and policy makers to better understand the needs and challenges faced by people living with BPD when transitioning to and utilising telehealth services. Addressing these shortcomings would improve telehealth service delivery acceptability and outcomes. One recommendation is for mental health clinicians engaging with clients for treatment via telehealth, to consider applying a checklist that asks clients about the challenges and concerns they might have. This may assist in deciding whether and when telehealth is suitable for individual clients, particularly given the focus of this study, if they have BPD-associated difficulties. Other challenges such as limited access to and proficiency with telehealth technology could potentially be addressed by healthcare services through the provision of training and guidance. Difficulties associated with attention span and screen fatigue are widely experienced. One simple solution could be to implement shorter duration telehealth sessions more frequently. In regions that experience poor internet coverage, additional resourcing and the enforcement of minimum connectivity standards could be implemented by state and national health departments. Supporting telehealth should be a priority as it greatly improves equity of access to treatment services for people living in regional and remote locations.

A US survey of mental health clinicians and service providers concluded that there was a high likelihood that telehealth use would continue post-pandemic.^19^ This is also highly likely in Australia, given telehealth benefits such as improved access to services and provision of person-centred care. Given the low pre-pandemic engagement of this sample of community participants with public mental health services, initiatives to increase resourcing and staff training to improve telehealth services constitutes a fair and effective way to improve access and availability of mental health services across the public health sector. Mobility is often a barrier when it comes to accessing healthcare. Telehealth transcends this issue by improving the availability of therapy to people who are less able to travel to appointments, who live in remote areas, have physical constraints including disability, or comorbid mental health issues that prevent them from leaving home. From a policy-making perspective, establishing clear guidelines that help to determine the suitability of telehealth for meeting the person's needs is essential. Such a process should be revisited regularly to confirm appropriateness of telehealth services and mitigate risk in people living with BPD.

### Limitations

The findings from this study reflect self-report; data were collected via an anonymous online survey. The online nature of the survey likely restricted participation to people with familiarity and access to digital services. Although the study was promoted through channels most likely be accessed by people with a diagnosis of BPD, there was no formal method of diagnosis; participants identified themselves as having a diagnosis of BPD.

### Conclusion

This article reported the experience of telehealth for mental health service users with lived experience of BPD during the COVID-19 pandemic. The study findings highlighted a variety of positive and negative experiences that provide insight into the ways that telehealth can be most effectively utilised into the future. While the majority of participants found telehealth somewhat benefitted their mental health, they also reported challenges which raise concerns regarding the utility and effectiveness of telehealth. The appropriateness of providing clinican services via telehealth should be regularly assessed for clients who experience BPD-associated symptoms such as emotion dysregulation, suicidality, and fear of abandonment.

## Supplemental Material

sj-docx-1-dhj-10.1177_20552076231169824 - Supplemental material for Mental health support for and telehealth use by Australians living with borderline personality disorder during the onset of the COVID-19 pandemic: A national study Click here for additional data file. Supplemental material, sj-docx-1-dhj-10.1177_20552076231169824 for Mental health support for and telehealth use by Australians living with borderline personality disorder during the onset of the COVID-19 pandemic: A national study by Parvaneh Heidari, Jillian H Broadbear, Rita Brown, Nitin P Dharwadkar and Sathya Rao in DIGITAL HEALTH
