# Supplementary material for: Mental health support for and telehealth use by Australians living with borderline personality disorder during the onset of the COVID-19 pandemic: A national study
Source: Digit Health. 2023 May 4;9:20552076231169824. doi: 10.1177/20552076231169824 (PMC10164265; doi:10.1177/20552076231169824)
Supplement: sj-docx-1-dhj-10.1177_20552076231169824 - Supplemental material for Mental health support for and telehealth use by Australians living with borderline personality disorder during the onset of the COVID-19 pandemic: A national study [file sj-docx-1-dhj-10.1177_20552076231169824.docx]

# Supplementary material.

Demographic characteristics of Australians living with borderline personality disorder.

| **States (n=169)** | N | % |
| --- | --- | --- |
| Victoria | 71 | 42.0 |
| New South Wales | 37 | 21.9 |
| Queensland | 18 | 10.7 |
| South Australia | 19 | 11.2 |
| Australian Capital Territory | 11 | 6.5 |
| Western Australia | 11 | 6.5 |
| Tasmania | 2 | 1.2 |
| **Gender (n=169)** |  |  |
| Female | 152 | 89.9 |
| Male | 8 | 4.7 |
| Non-binary | 7 | 4.1 |
| Transgender/ gender fluid | 2 | 1.2 |
| **Age (n=169)** |  |  |
| 18-27 | 34 | 20.1 |
| 28-37 | 59 | 34.9 |
| 38-47 | 40 | 23.7 |
| 48-57 | 22 | 13.0 |
| 58-67 | 11 | 6.5 |
| 68 and over | 1 | 0.6 |
| Prefer not to answer | 2 | 1.2 |
| **Comorbidities (n=161)** |  |  |
| Depression | 146 | 96.4 |
| Bipolar disorder | 26 | 15.4 |
| Schizophrenia or other psychotic disorder | 3 | 1.8 |
| Anxiety | 133 | 78.7 |
| Eating disorder | 31 | 18.3 |
| Substance Use Disorder | 20 | 11.8 |
| Other Personality Disorder | 14 | 8.3 |
| Chronic Pain | 36 | 21.3 |
| Diabetes | 11 | 6.5 |
| Heart condition | 8 | 4.7 |
| PTSD or CPTSD | 14 | 8.3 |
| **Living arrangement (n=169)** |  |  |
| I live alone | 39 | 23.1 |
| I lived with my parents and/or siblings | 28 | 16.6 |
| I lived with my partner (no children) | 32 | 18.9 |
| I lived with my partner and children/children | 44 | 26.0 |
| I lived with one or more friends | 4 | 2.4 |
| I shared my house with housemates | 13 | 7.7 |
| Other | 8 | 4.7 |
| Prefer not to answer | 1 | 0.6 |
| **Having pets (n=169)** | 127 | 75.1 |
| **Has your occupation changed during the restrictions? (n=141)** |  |  |
| I worked as usual at my workplace | 23 | 13.6 |
| I shifted to working from home | 28 | 16.6 |
| I changed to supervising my children with their home-schooling  (or providing child-care) | 4 | 2.4 |
| I began studying remotely | 22 | 13.0 |
| I didn't have a formal occupation prior to the restrictions | 48 | 28.4 |
| I lost some of my employment | 13 | 7.7 |
| I lost all of my employment | 21 | 12.4 |
| I was employed in a new job | 10 | 5.9 |
| **What mental health services and support were you receiving prior to the restrictions? (n=165)** |  |  |
| Prevention and Recovery Care (PARC) | 5 | 3.0 |
| Crisis Assessment and Treatment Team (CATT) | 18 | 10.7 |
| Drug and alcohol services | 7 | 4.1 |
| Private psychologist | 77 | 45.6 |
| Private psychiatrist | 46 | 27.2 |
| Case management | 24 | 14.2 |
| Public psychologist | 30 | 17.8 |
| Public psychiatrist | 19 | 11.2 |
| Mental Health nurse through GP Clinic | 20 | 11.8 |
